# Short versus Long Gonadotropin-Releasing Hormone Analogue Suppression Protocols in IVF/ICSI Cycles in Patients of Various Age Ranges

**DOI:** 10.1371/journal.pone.0133887

**Published:** 2015-07-24

**Authors:** Jianping Ou, Weijie Xing, Yubin Li, Yanwen Xu, Canquan Zhou

**Affiliations:** 1 Center for Reproductive Medicine, The Third Affiliated Hospital of Sun Yat-sen University, Guangzhou, China; 2 Center for Reproductive Medicine, The First Affiliated Hospital of Sun Yat-sen University, Guangzhou, China; Institute of Zoology, Chinese Academy of Sciences, CHINA

## Abstract

**Objective:**

To compare the two GnRH-a protocols (long GnRH-a protocol and short GnRH-a protocol) for ovarian stimulation in IVF/ICSI cycles in patients of various age ranges.

**Methods:**

A total of 5662 IVF-ET/ICSI cycles from 2010 to 2013 were retrospectively identified. The cycles were divided into two groups: a long protocol group and short protocol group. In each group, the patients were divided into four age ranges: <31 years, 31 to 35 years, 36 to 40 years, and >40 years. The duration of stimulation, total dose of Gn, implantation rate and pregnancy rate were compared.

**Results:**

The total dose of Gn was significantly higher, and the duration of stimulation was significantly longer, in the long protocol group than in the short protocol group for all age ranges (*P*<0.05). If the patients were of the same age range, the number of oocytes retrieved, MII oocytes, and high-quality embryos in the long protocol group were all significantly greater than those in the short protocol group (*P*<0.05). In the long protocol group, the clinical pregnancy rates of the four age ranges were 52.76%, 44.33%, 36.15% and 13.33%, respectively, which were significantly higher than those in the short protocol group (33.33%, 24.58%, 22.49% and 8.72%, respectively; *P*<0.05). The same trend was also found in the implantation rates of the four age ranges. As the age increased, the clinical pregnancy and implantation rates, as well as the number of oocytes retrieved, MII oocytes, and high-quality embryos, of the long protocol group significantly decreased (*P*<0.05).

**Conclusions:**

Our study demonstrated that regardless of patient age, the long protocol was superior to the short protocol in terms of the number of retrieved oocytes, as well as the implantation and pregnancy rates.

## Introduction

Controlled ovarian hyper-stimulation (COH) is a highly important component of in vitro fertilization-embryo transfer/intra-cytoplasmic sperm injection (IVF-ET/ICSI) cycles. Porter et al first reported that gonadotropins (Gn) with Gonadotropin-releasing hormone agonists (GnRH-a) were used for ovarian stimulation in IVF cycles [1]. Pituitary suppression was obtained by GnRH-a to prevent premature luteinization. There are two primary protocols used for this purpose: a long protocol and short protocol. In the long protocol, GnRH-a is commonly administered in the middle of the luteal phase of the previous cycle, whereas in the short protocol, GnRH-a is administered at the beginning of the menstruation cycle and continues until administration of hCG.

The long GnRH-a protocol is the most popular regimen in IVF-ET/ICSI treatment, and excellent results have been obtained [2]. The short GnRH-a protocol was suggested as an alternative protocol. Because GnRH agonist can induce an initial “flare-up” effect, the short GnRH-a protocol is usually provided to poor responders to avoid excessive pituitary suppression [3]. There is no consensus on which protocol is the best treatment for all patients. Many studies do not demonstrate any significant differences between the two protocols. Two meta-analyses exhibited different results. The data in one meta-analysis reported that the long protocol was more effective than the short protocol in terms of oocyte retrieval and clinical pregnancy rates [2]. However, the other meta-analysis concluded that there was no difference between the short and long protocols [4].

The aim of this study was to compare the two protocols in terms of stimulation characteristics and IVF/ICSI outcomes in patients of various age ranges.

## Materials and Methods

A total of 5662 IVF-ET/ICSI cycles from 2010–2013 were retrospectively identified. The cycles were divided into two groups: a long protocol group and a short protocol group. In each group, the patients were divided into four age ranges: <31 years, 31 to 35 years, 36 to 40 years, and >40 years. This retrospective study was approved by the Third Affiliated Hospital of Sun Yat-Sen University Reproductive Medicine Ethic Committee. The patient records/information was anonymized and de-identified prior to analysis.

We have the standard operation procedure (SOP) for all the physicians and embryologists. The patients in the long protocol group underwent down-regulation by receiving 1.0 mg-1.3 mg Triptorelin (3.75 mg Gonapeptyl; Ferring) during the mid-luteal phase of the preceding cycle. Complete pituitary suppression was confirmed by a serum E_2_ level <30 pg/mL and serum LH level <2 mIU/mL[5]. Recombinant FSH (Gonal-F, Serono, Switzerland) and/or hMG (LiZHu, China) were used at doses ranging between 100 IU/day and 450 IU/day in accordance with body mass index, patient age, and size and number of follicles. The dosage of FSH and hMG was adjusted according to ovarian response, which was assessed by ultrasound and serum E_2_ levels. Recombinant hCG (Serono, Switzerland) was given to trigger follicle maturation when at least two follicles reached a mean diameter of 18 mm. Oocyte retrieval was performed transvaginally 34–36 hours after hCG injection.

In the short protocol group, a dose of Triptorelin (0.1 mg Gonapeptyl; Ferring) was administered beginning on day 3 of the menstrual cycle. After 1 to 2 days, ovarian stimulation commenced with 100 IU-450 IU rFSH (Gonal-F; Serono, Switzerland) and/or hMG daily. The dosage of FSH and hMG was adjusted according to ovarian response. Oocyte retrieval was performed 34–36 hours after hCG trigger.

Embryo transfers were performed 3 to 5 days later using ultrasound guidance. Pregnancy was diagnosed by a rising concentration of serum β-hCG, which was tested 14 days after ET. Clinical pregnancy was defined as the presence of a gestational sac during vaginal ultrasound examination. Miscarriage was defined as pregnancy loss before 28 weeks.

The SPSS statistical software package (version 11.0) was used for statistical analysis. Values were expressed as the mean ± SD. The unpaired Student’s t-test was used to compare means of the two groups. One-way analysis of variance (ANOVA) with a post hoc test using Fisher’s Protected Least Significant Difference (PLSD) was used to compare multiple means from various groups. χ^2^-test was used to compare categorical variables. *P* < 0.05 was considered statistically significant.

## Results

A total of 5662 IVF-ET/ICSI cycles were retrospectively studied. The patient demographic variables are listed in Table 1. In each age range, the two groups (long protocol group and short protocol group) were similar in terms of body mass index and proportion of primary infertility.

**Table 1:** Demographic characteristics of patients in long and short protocol group.

Variable	Groups		*P*
	Long Protocol Group	Short Protocol Group	
No. of cycles			
<31 years	2140	87	*/*
31–35 years	1906	179	*/*
36–40 years	816	249	*/*
>40 years	90	195	*/*
Body Mass Index (kg/m^2^)			
<31 years	20.68 ± 2.47	20.68 ± 2.36	ns
31–35 years	21.16 ± 2.52	21.28 ± 2.61	ns
36–40 years	21.75 ± 2.59	21.87 ± 2.37	ns
>40 years	22.17 ± 2.35	22.29 ± 3.06	ns
Primary infertility (n, %)			
<31 years	59.81% (1280/2140)	54.02% (47/87)	ns
31–35 years	40.89% (875/2140)	45.81% (82/179)	ns
36–40 years	37.50% (306/816)	30.92% (77/249)	ns
>40 years	34.44% (31/90)	23.08% (45/195)	ns
Duration of infertility (years)			
<31 years	3.43 ± 2.03	3.36 ± 2.45	ns
31–35 years	4.57 ± 2.63	5.54 ± 2.87	ns
36–40 years	6.43 ± 3.93	6.91 ± 4.47	<0.05
>40 years	7.37 ± 5.06	7.23 ± 5.09	ns
No. of IVF cycles (n, %)			
<31 years	51.73% (1107/2140)	60.92% (53/87)	ns
31–35 years	42.08% (802/1906)	68.16% (122/179)	<0.05
36–40 years	58.82% (480/816)	55.02% (137/249)	ns
>40 years	46.67% (42/90)	58.97% (115/195)	ns

Note: NS = not statistically significant. Values presented as mean ± SD unless otherwise specified.

**Fig 1:**
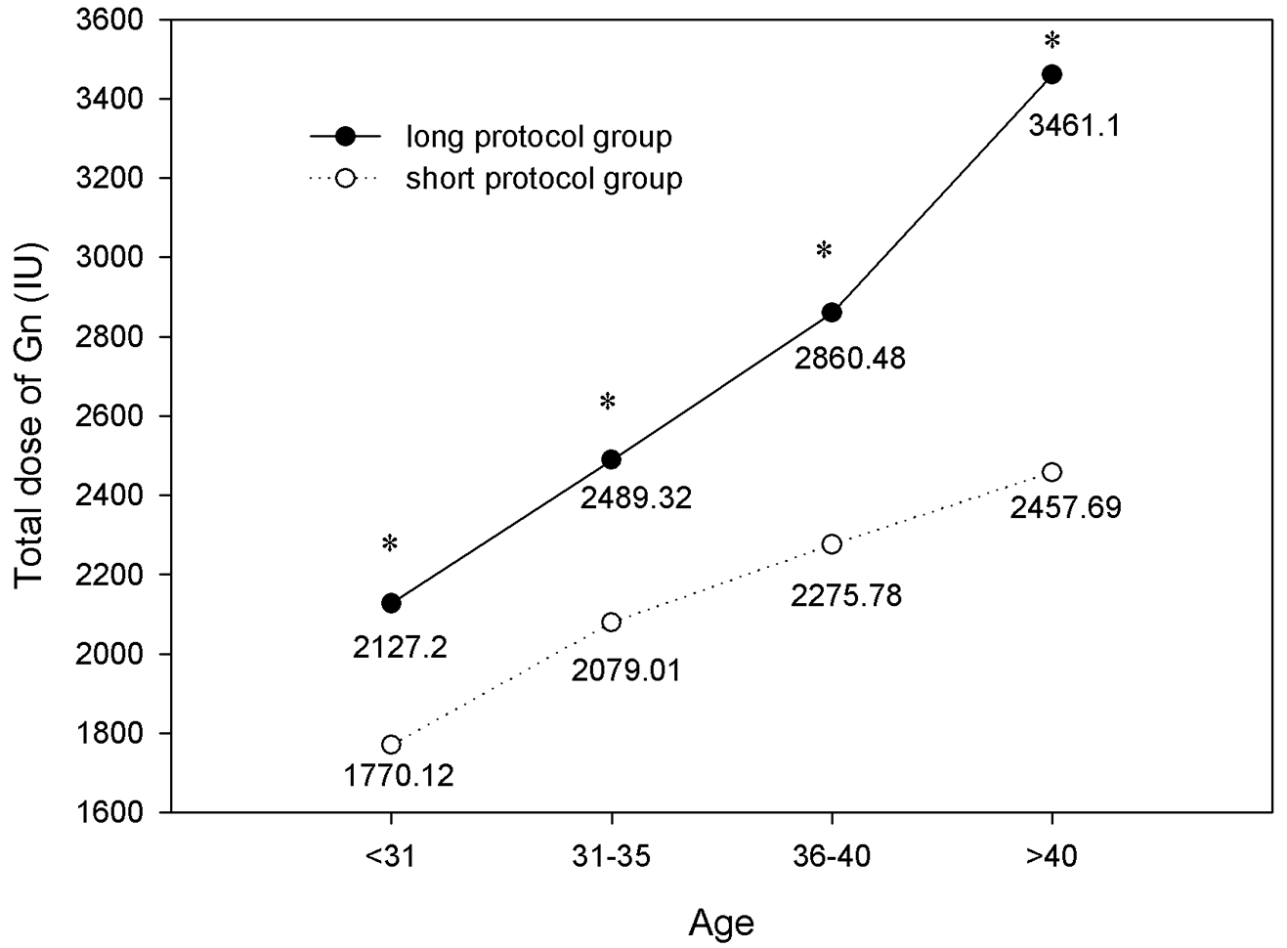
Comparison of total dose of Gn. The total dose of Gn was significantly higher in the long protocol group than in the short protocol group for all age ranges (*P*<0.05). As age increased, more gonadotropins were consumed (*P*<0.05). * *P*<0.05 versus the short protocol group.

**Fig 2:**
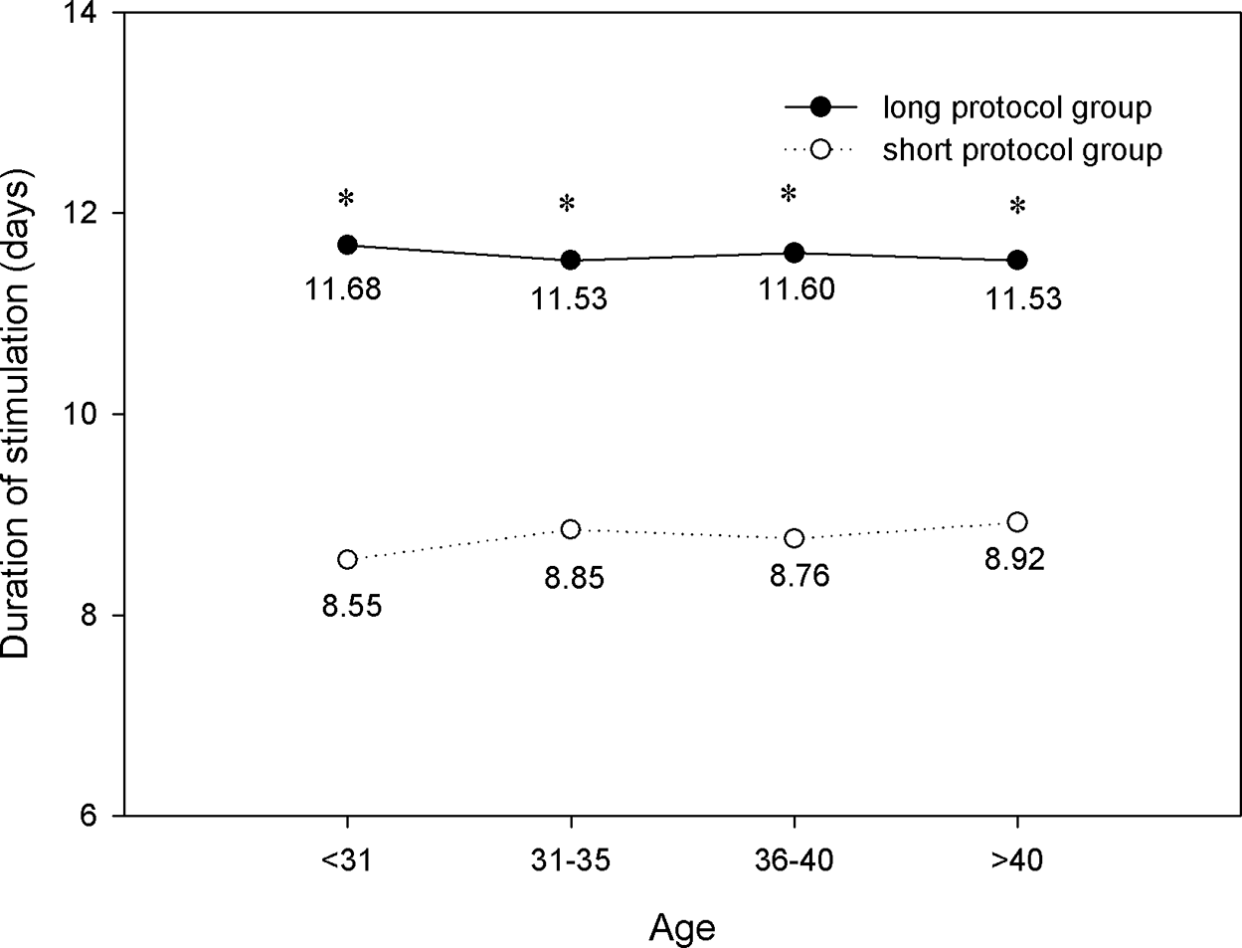
Comparison of stimulation duration. The duration of stimulation was significantly longer in the long protocol group than that in the short protocol group for all age ranges (*P*<0.05). As age increased, the durations remained the same. * *P*<0.05 versus the short protocol group.

As shown in Figs 1 and 2, the total dose of Gn was significantly higher, and the duration of stimulation was significantly longer, in the long protocol group than in the short protocol group for all age ranges (*P*<0.05). As age increased, more gonadotropins were consumed (*P*<0.05), but the durations remained the same.

**Fig 3:**
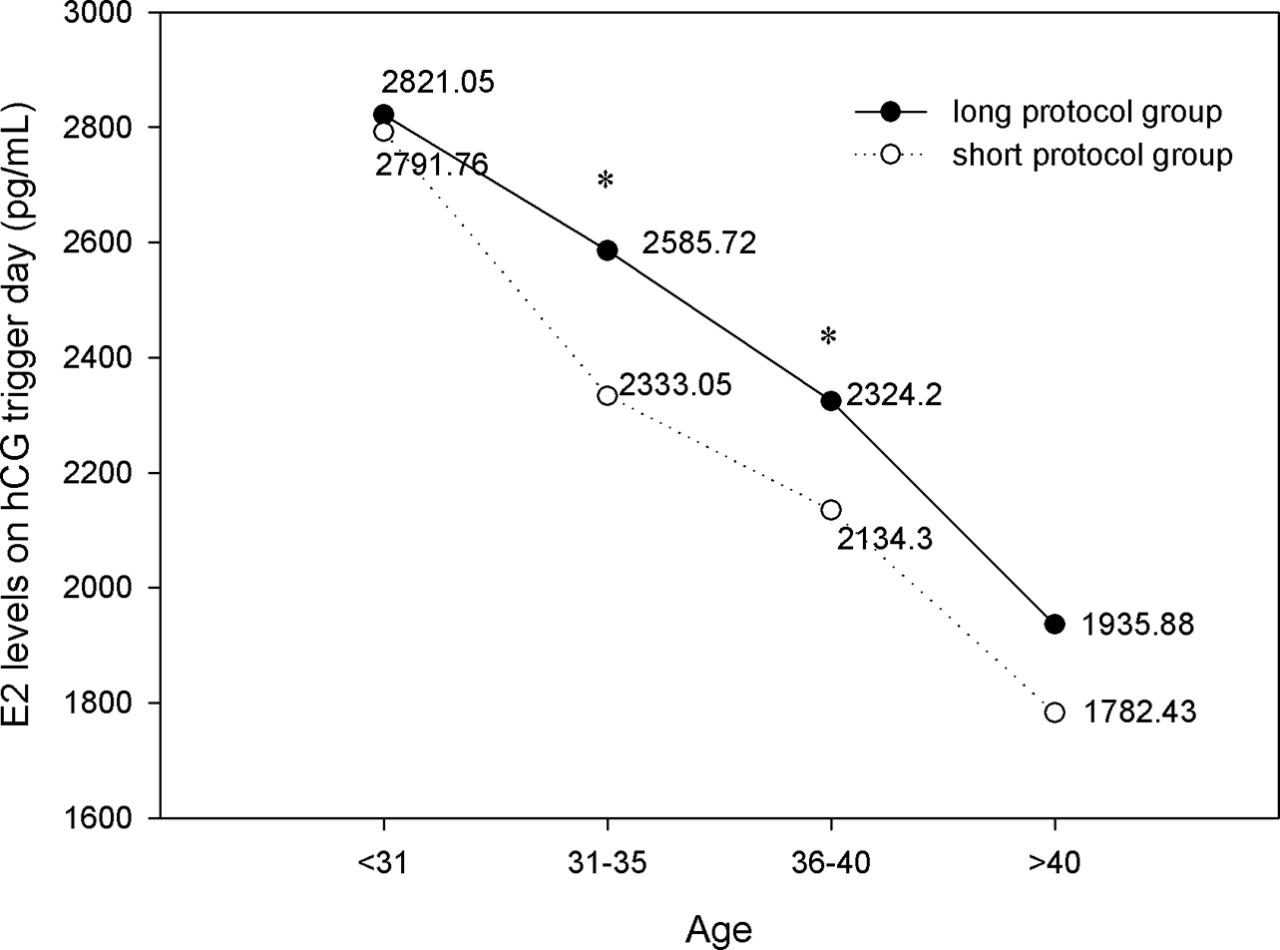
Comparison of E_2_ levels on hCG trigger day. The E_2_ levels on hCG trigger day of the two groups were similar in two age ranges (age < 31 and age >40); In the other two age ranges, the E_2_ levels were 2585.72 pg/mL and 2324.2 pg/mL in the long protocol group, and these values were significantly higher than those in the short protocol group (2333.05 pg/mL and 2134.3 pg/mL, respectively; *P*<0.05). * *P*<0.05 versus the short protocol group.

As shown in Fig 3, the E_2_ levels on hCG trigger day of the two groups were similar in two age ranges (age < 31 and age >40); In the other two age ranges, the E_2_ levels were 2585.72 ± 1319.10 pg/mL and 2324.20 ± 1281.79 pg/mL in the long protocol group, and these values were significantly higher than those in the short protocol group (2333.05 ± 1276.37 pg/mL and 2134.30 ± 1224.02 pg/mL, respectively; *P*<0.05).

**Fig 4:**
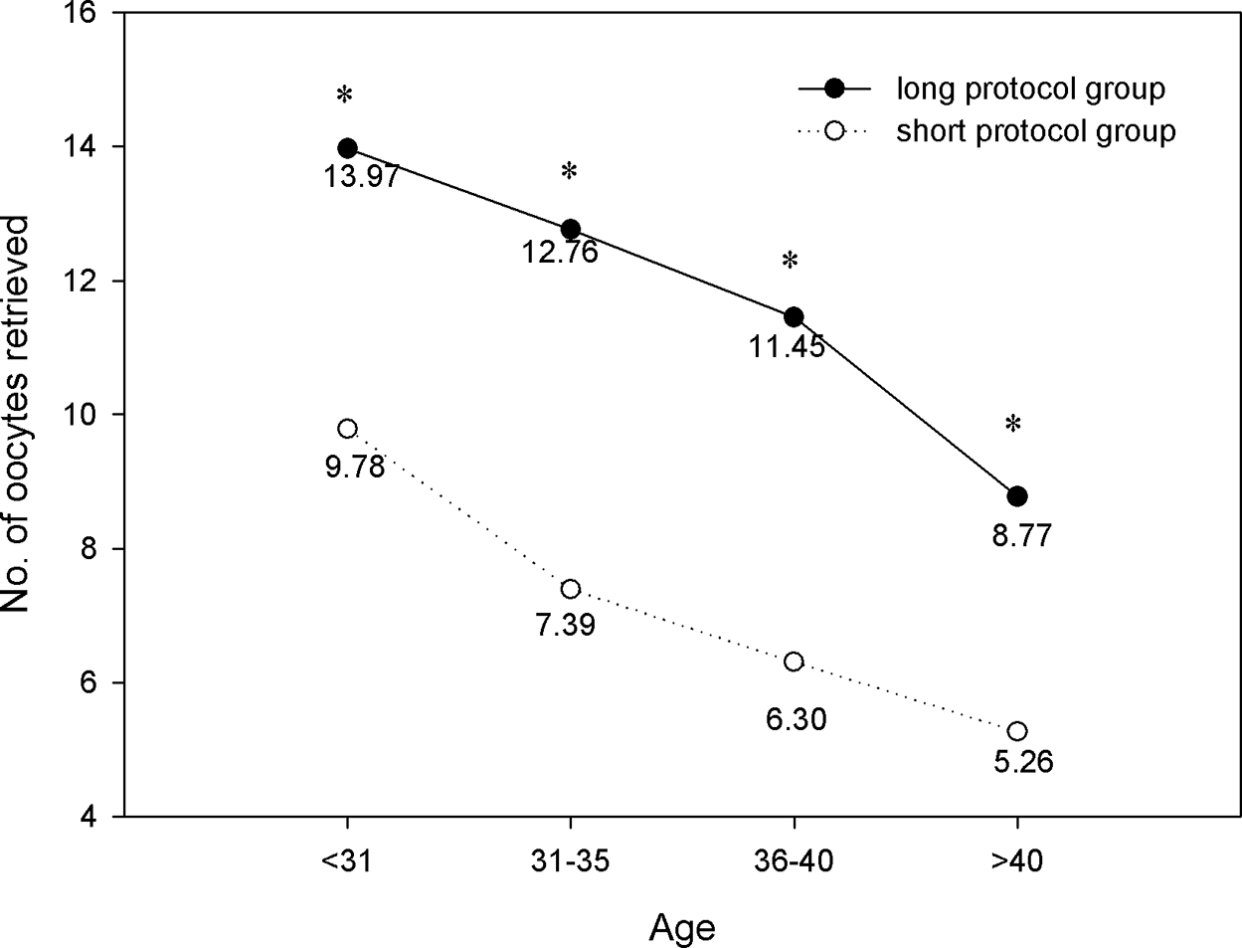
Comparison of No. of oocytes retrieved. Within each age range, the number of retrieved oocytes in the long protocol group was significantly greater than those in the short protocol group (*P*<0.05). They decreased with increasing age, and the differences were significant according to one-way ANOVA analysis (*P*<0.05). * *P*<0.05 versus the short protocol group.

**Fig 5:**
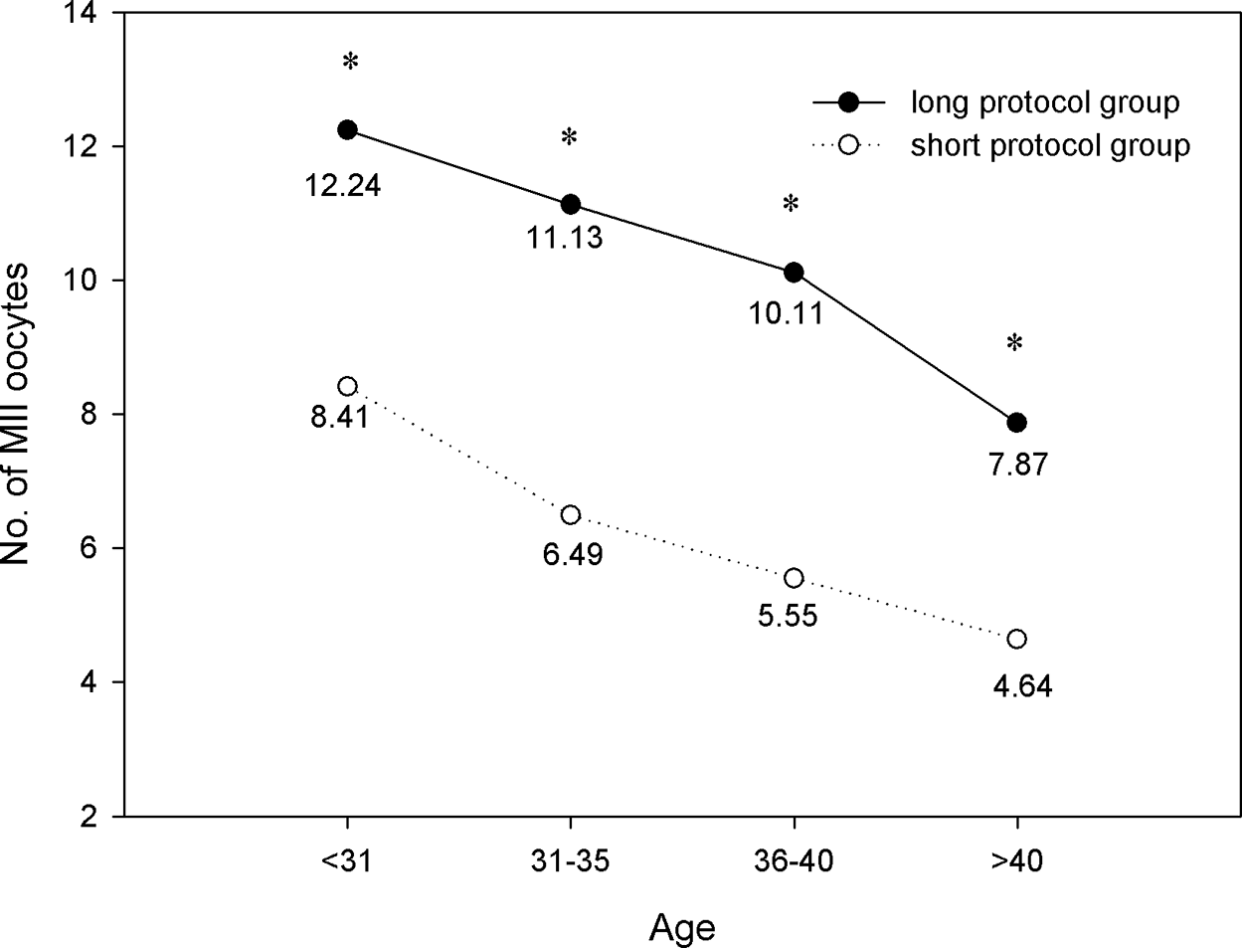
Comparison of No. of MII oocytes. Within each age range, the number of MII oocytes in the long protocol group was significantly greater than that in the short protocol group (*P*<0.05). This value significantly decreased with increasing age (*P*<0.05). * *P*<0.05 versus the short protocol group.

**Fig 6:**
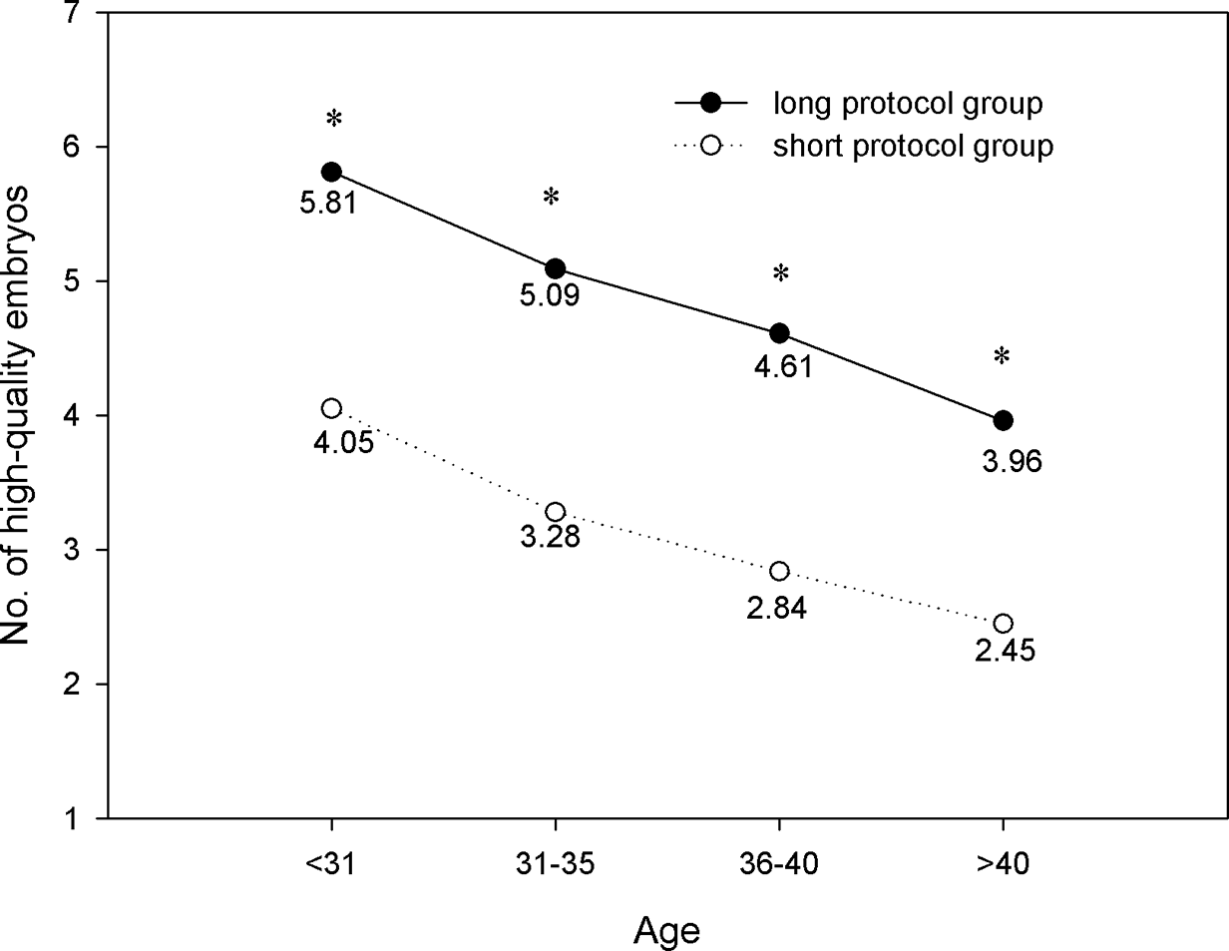
Comparison of No. of high-quality embryos. The number of high-quality embryos in the long protocol group was significantly greater than that in the short protocol group for all age ranges (*P*<0.05). This value significantly decreased with increasing age (*P*<0.05). * *P*<0.05 versus the short protocol group.

**Fig 7:**
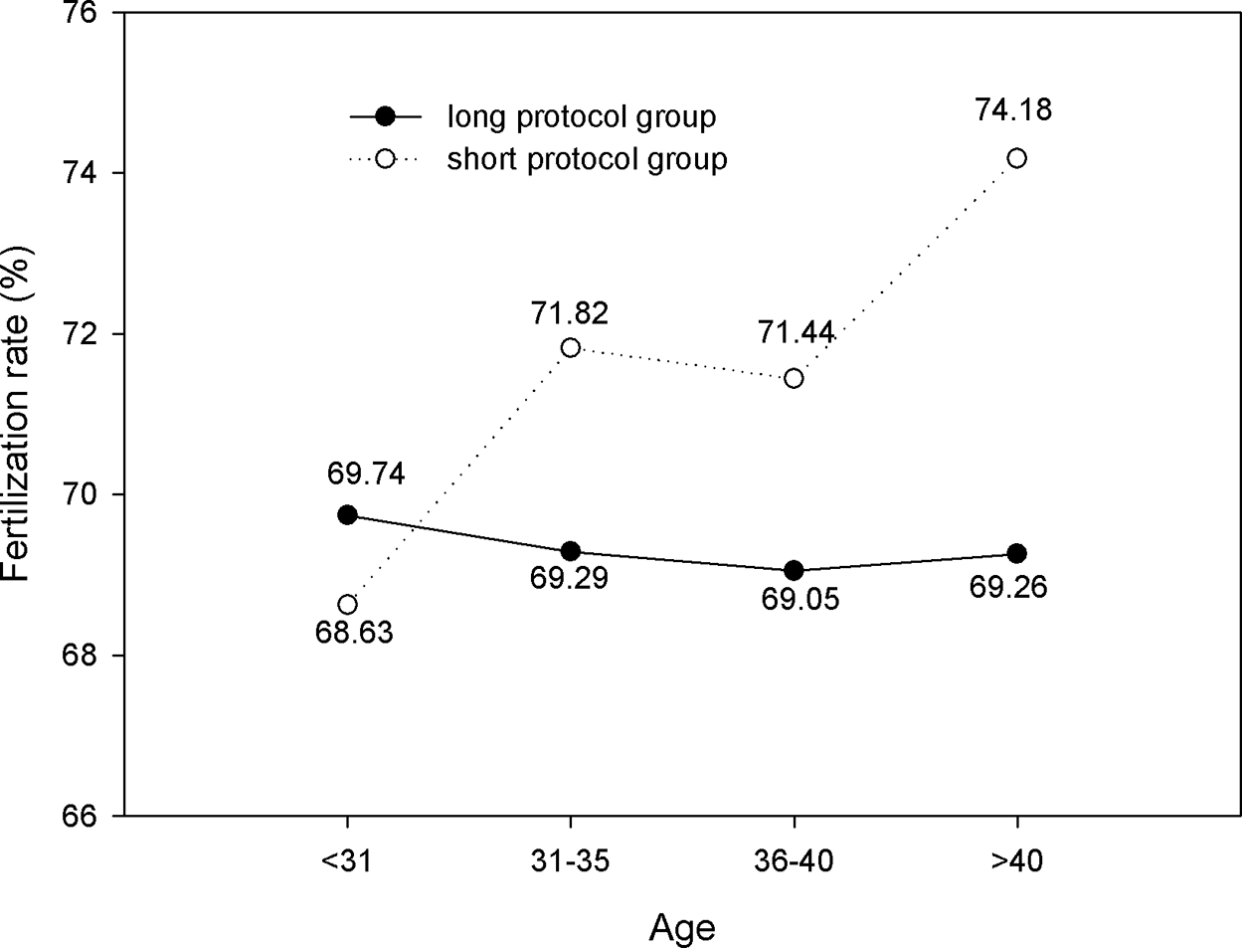
Comparison of fertilization rate. In those younger than 31 years, the fertilization rate of the long protocol group was 69.74%, which was slightly higher than that of the short protocol group (68.63%). However, in patients older than 31 years, the fertilization rate of the long protocol group was slightly lower than that of the short protocol group.

The number of oocytes retrieved, MII oocytes, and high-quality embryos are shown in Figs 4–6. We found that within each age range, the number of oocytes retrieved, MII oocytes, and high-quality embryos in the long protocol group were all significantly greater than those in the short protocol group (*P*<0.05). As expected, they all decreased with increasing age, and the differences were significant according to one-way ANOVA analysis (*P*<0.05). As shown in Fig 7, we observed that in the age range of less than 31 years, the fertilization rate of the long protocol was 69.74%, which was slightly higher than that of the short protocol (68.63%). However, in patients older than 31 years, the fertilization rates of the long protocol group were all slightly lower than those of the short protocol group.

**Fig 8:**
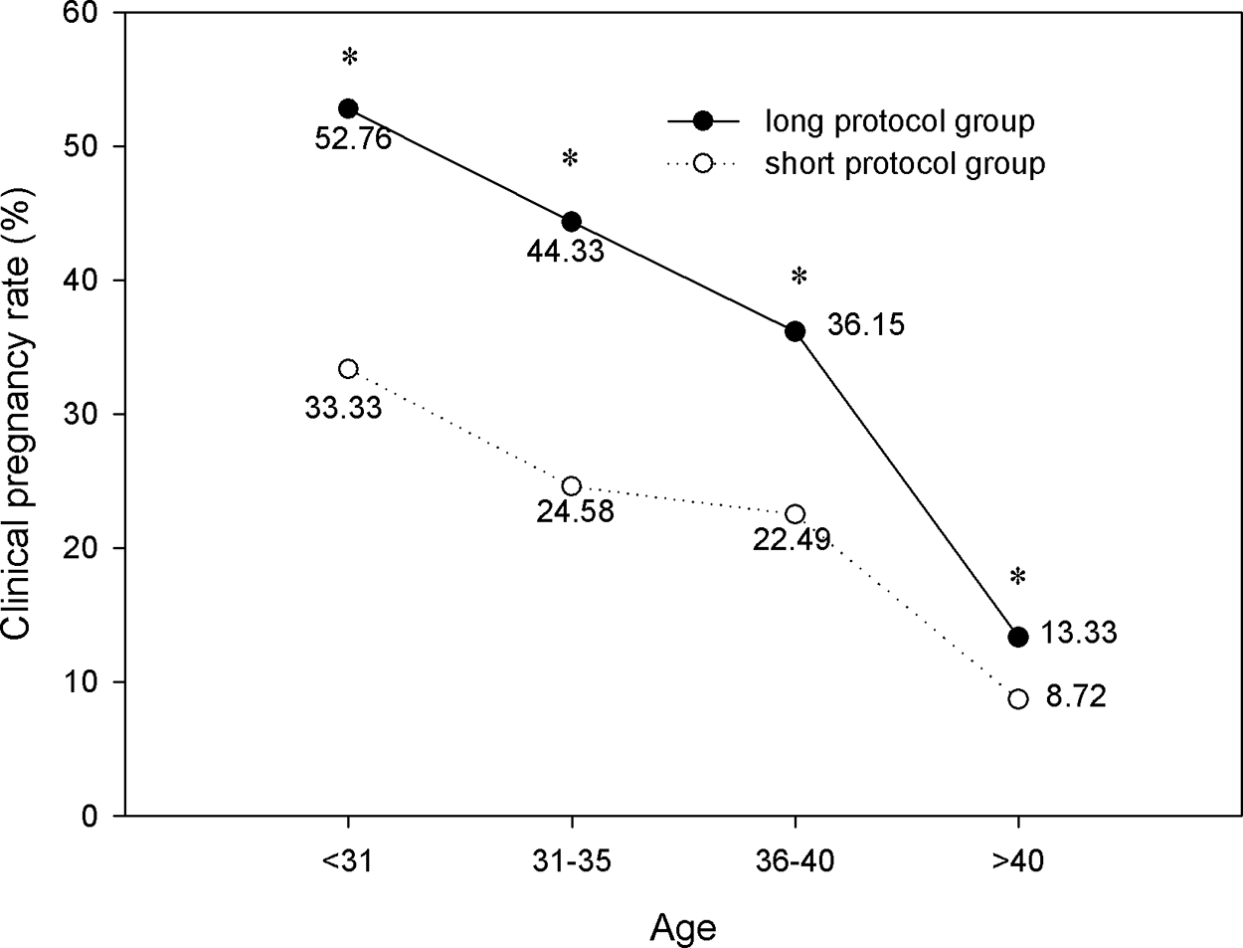
Comparison of clinical pregnancy rate. In the long protocol group, the clinical pregnancy rates of the four age ranges were 52.76%, 44.33%, 36.15% and 13.33%, respectively, which were significantly higher than those in the short protocol group (33.33%, 24.58%, 22.49% and 8.72%, respectively; *P*<0.05). As aged increased, the clinical pregnancy rate in the long protocol group significantly decreased (*P*<0.05). * *P*<0.05 versus the short protocol group.

**Fig 9:**
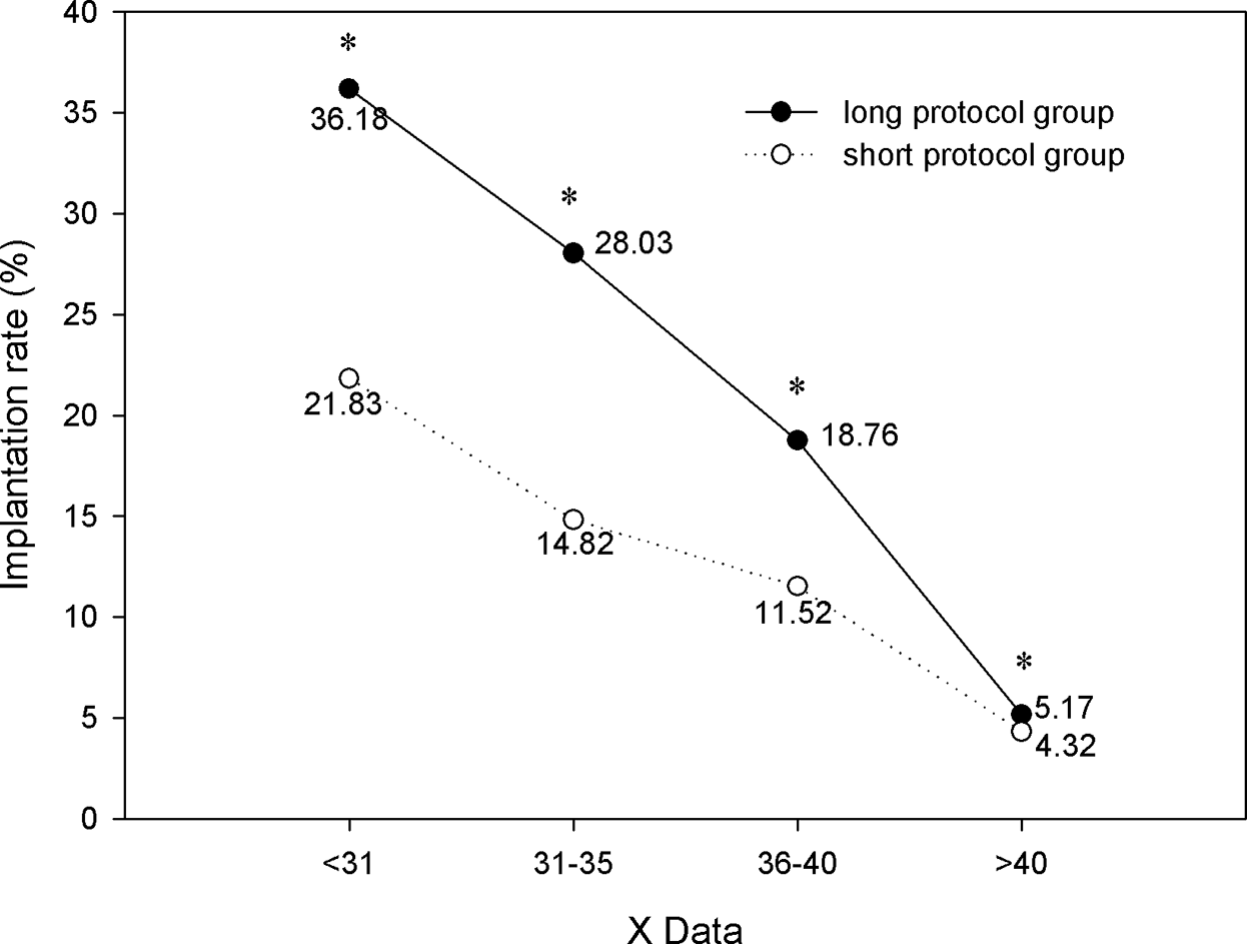
Comparison of implantation rate. In the long protocol group, the implantation rates of the four age ranges were significantly higher than those in the short protocol group (P<0.05). As aged increased, the implantation rates of the long protocol group significantly decreased (P<0.05). * *P*<0.05 versus the short protocol group.

Fig 8 demonstrated that in the long protocol group, the clinical pregnancy rates of the four age ranges were 52.76%, 44.33%, 36.15% and 13.33%, respectively, which were significantly higher than those in the short protocol group (33.33%, 24.58%, 22.49% and 8.72%, respectively; *P*<0.05). The same trend was also found in the implantation rates of the four age ranges (Fig 9). The χ^2^-test demonstrated the following results: (1) As age increased, the clinical pregnancy rate and implantation rate of the long protocol group significantly decreased (*P*<0.05; Fig 8); (2) In the short protocol group in patients older than 40 years, the pregnancy rate was 8.72%, which was significantly lower than the pregnancy rate of the other three age ranges (33.33%, 24.58% and 22.49%, respectively; *P*<0.05; Fig 8); (3) In the short protocol group in patients older than 40 years, the implantation rate was 4.32%, which was significantly lower than the implantation rate of the other three age ranges (21.83%, 14.82% and 11.52%, respectively; *P*<0.05; Fig 9).

**Fig 10:**
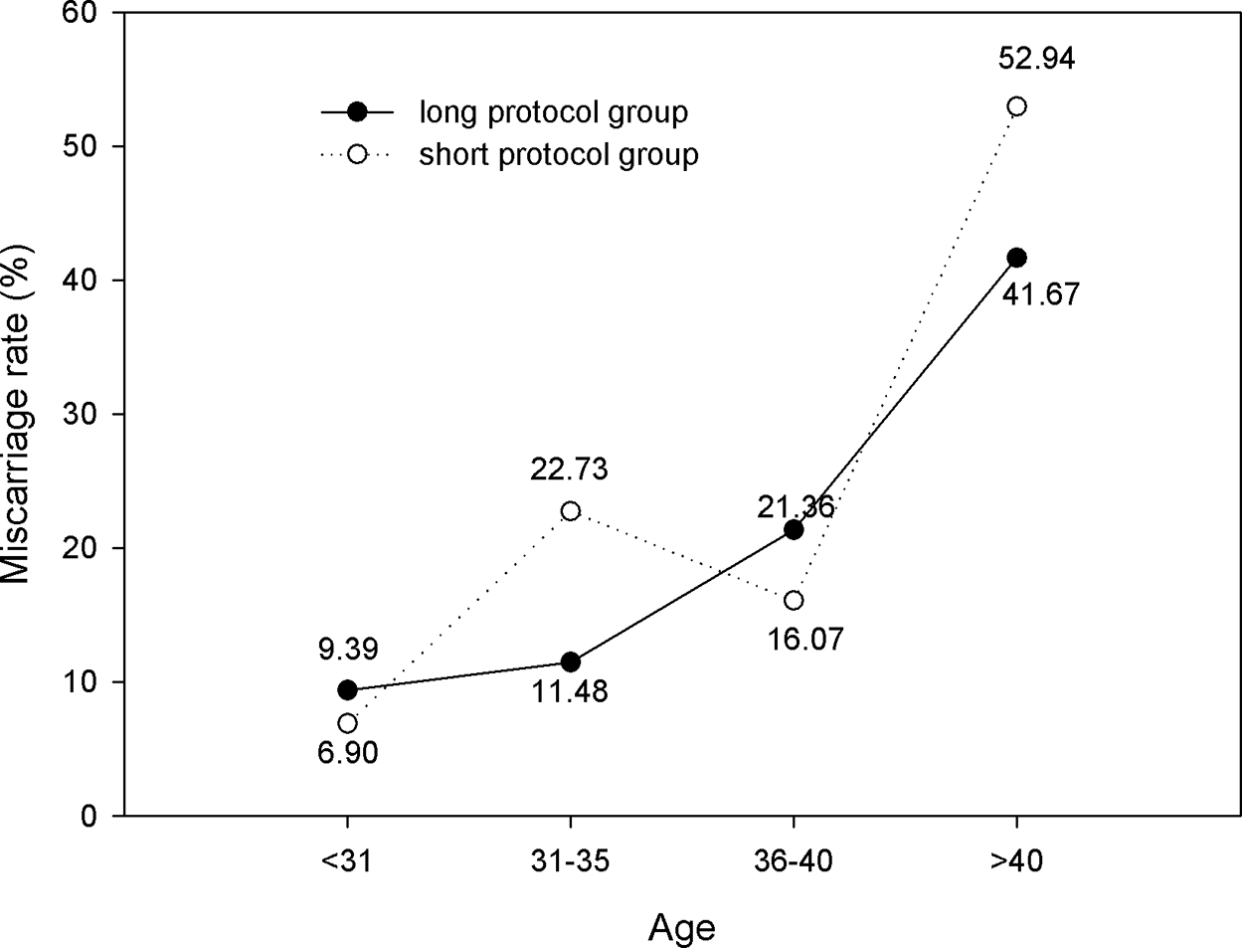
Comparison of miscarriage rate. For all age ranges, there were no significant differences in miscarriage rates between the two groups. In the short protocol group in patients older than 40 years, the miscarriage rate was 52.94%, which was significantly higher than that of the other three age ranges (6.90%, 22.73% and 16.07%, respectively; *P*<0.05).

In all age ranges, there were no significant differences in miscarriage rates between the two groups (Fig 10). Fig 10 also indicates that in the short protocol group in patients older than 40 years, the miscarriage rate was 52.94%, which was significantly higher than the miscarriage rate of the other three age ranges (6.90%, 22.73% and 16.07%, respectively; *P*<0.05).

## Discussion

In the late 1980s, GnRH-a was first used in COH protocols to prevent premature luteinization [6]. During the past decade, several modalities have been introduced to improve ART outcomes. These modalities include the following: (1) different protocols were used, such as long GnRH agonist protocol, short GnRH agonist protocol and GnRH antagonist protocol; (2) variations in the type, dose, and timing of gonadotropins, or GnRH analogues (agonists and antagonists), for decreasing ovarian suppression and cycle cancellation rates; (3) the use of oral contraceptive (OC) pills, growth hormone, and testosterone as adjuvant therapies to enhance oocyte yield [7,8,9,10]. However, there were still many uncertainties about the optimal protocol for ovarian stimulation in in vitro fertilization (IVF) [6].

It has been supposed that a long GnRH agonist regimen in IVF may lead to ovarian over-suppression. In this study, our results showed that there was significantly more gonadotropin consumption and a longer duration of stimulation in the long protocol group compared with the short protocol group in all four age ranges. Ovarian over-suppression may be the cause. We also found that in patients of the same age range, the number of oocytes retrieved, MII oocytes, and high-quality embryos in the long protocol group were all significantly greater than those in the short protocol group. These findings were consistent with previous studies. Recently, a retrospective study showed that the number of oocytes and mature oocytes were significantly higher with the long GnRH agonist regimen compared with the short GnRH agonist mini-dose regimen [11]. By inducing hypophyseal desensitization with the long GnRH agonist regimen, the follicle synchronicity was better than that with the short GnRH agonist regimen [12].

Fig 8 demonstrates that in the long protocol group, the clinical pregnancy rates of the four age ranges were 52.76%, 44.33%, 36.15% and 13.33%, respectively, which were significantly higher than those in the short protocol group (33.33%, 24.58%, 22.49% and 8.72%, respectively; *P*<0.05). The same trend was also found in the implantation rates of the four age ranges (Fig 9). Several reasons can explain why the long protocol was better: (1) The long protocol, starting in the mid-luteal phase, results in a better follicular synchronization, in turn leading to an increase in size of the follicle cohort recruited for the cycle. And an increased length of stimulation might allow additional growing follicles to enter in the cohort of stimulated follicles. So, it can promote better follicle recruitment and more oocytes harvested. (2) Our results showed that within each age range, the number of oocytes retrieved, MII oocytes, and high-quality embryos in the long protocol group were all significantly greater than those in the short protocol group (P<0.05). The quality of transferred embryos was better in the long protocol group. (3) The receptivity of the endometrium may be more suitable for embryo implantation with the complete suppression and lower serum LH level in the long protocol. Previous studies have showed that LH, in addition to its well-known effects on the ovary, may exert direct effects on the endometrium[13]. In a recent study, their results suggested that lower serum LH levels in the long-acting GnRHa protocol might play a beneficial role for endometrial receptivity. Moreover, in that study, the endometrial thickness was significantly higher in long-acting GnRHa group than that in short GnRHa group[14].

The short protocol seemed to be an efficient and cost-effective protocol for the poor responders. The short GnRH agonist regimen was considered an alternative for poor responders to improve outcomes by avoiding excessive pituitary suppression and taking advantage of the initial flare effect of the GnRH agonist [3,15]. However, in our study, the long protocol was more effective than the short protocol in terms of number of retrieved oocytes and implantation and pregnancy rates, even in patients older than 40 years. These findings demonstrated that the long protocol was superior to the short protocol for all age ranges, even in patients older than 40 years.

We also determined that the number of retrieved oocytes, MII oocytes, and high-quality embryos, as well as the implantation and clinical pregnancy rates, decreased with increasing age in the two groups. During the past 10 years, many markers of ovarian reserve have also been used to predict the outcomes of ART. These markers include basal FSH, estradiol, inhibin, AMH and others [16,17]. However, age is the key factor in ART outcomes. Normal ovarian reserve in women older than 40 years do not predict high pregnancy rates [16]. James P. Toner decided that if some eggs were obtained, the pregnancy rates of young women with abnormal ovarian reserve were typically higher than those in older women with normal ovarian reserve.

## Conclusions

Our study demonstrated that regardless of patient age, the long protocol was superior to the short protocol in terms of number of retrieved oocytes and implantation and pregnancy rates.

## Supporting Information

S1 File All the outputs of the comparison of two protocols analysed by the SPSS statistical software package (version 11.0). (PDF)
