# Supplementary material for: Short versus Long Gonadotropin-Releasing Hormone Analogue Suppression Protocols in IVF/ICSI Cycles in Patients of Various Age Ranges
Source: PLoS One. 2015 Jul 24;10(7):e0133887. doi: 10.1371/journal.pone.0133887 (PMC4514806; doi:10.1371/journal.pone.0133887)
Supplement: S1 File — (PDF) [file pone.0133887.s001.pdf]

Variable	Groups		*P*
	Long Protocol Group	Short Protocol Group	
Total dose of Gn			
<31 years	2127.20±664.55	1770.12±746.28	<0.05
31-35 years	2489.32±723.40	2079.01±759.75	<0.05
36-40 years	2860.48±751.14	2275.78±706.88	<0.05
>40 years	3461.10±779.80	2457.69±676.51	<0.05
Duration of stimulation			
<31 years	11.68±1.89	8.55±2.05	<0.05
31-35 years	11.53±1.72	8.85±2.07	<0.05
36-40 years	11.60±1.70	8.76±2.00	<0.05
>40 years	11.53±1.50	8.92±1.90	<0.05
E_2 levels on hCG trigger day			
<31 years	2821.05±1302.86	2791.76±1411.31	ns
31-35 years	2585.72±1319.10	2333.05±1276.37	<0.05
36-40 years	2324.20±1281.79	2134.30±1224.02	<0.05
>40 years	1935.88±1194.47	1782.43±1014.35	ns
No. of oocytes retrieved			
<31 years	13.97±5.76	9.78±6.09	<0.05
31-35 years	12.76±5.83	7.39±4.64	<0.05
36-40 years	11.45±5.73	6.30±4.15	<0.05
>40 years	8.77±5.04	5.26±3.22	<0.05
No. of MII oocytes			
<31 years	12.24±5.36	8.41±5.47	<0.05
31-35 years	11.13±5.35	6.49±4.19	<0.05
36-40 years	10.11±5.29	5.55±3.69	<0.05
>40 years	7.87±4.79	4.64±2.85	<0.05
No. of high-quality embryos			
<31 years	5.81±3.81	4.05±3.12	<0.05
31-35 years	5.09±3.47	3.28±2.41	<0.05
36-40 years	4.61±2.89	2.84±1.88	<0.05
>40 years	3.96±2.88	2.45±1.32	<0.05
